# Human enamel thickness and *ENAM* polymorphism

**DOI:** 10.1038/ijos.2016.1

**Published:** 2016-04-01

**Authors:** Diane M Daubert, Joanna L Kelley, Yuriy G Udod, Carolina Habor, Chris G Kleist, Ilona K Furman, Igor N Tikonov, Willie J Swanson, Frank A Roberts

**Affiliations:** 1 Department of Periodontics, University of Washington, Seattle, USA; 2 Center for Reproductive Biology and School of Biological Sciences, Washington State University, Pullman, USA; 3 Department of Oral and Maxillofacial Surgery, University of Texas Southwestern Medical Center, Dallas, USA; 4 Department of Genome Sciences, University of Washington, Seattle, USA

**Keywords:** enamel, genetics, polymorphisms, tooth, development

## Abstract

The tooth enamel development gene, enamelin (*ENAM)*, showed evidence of positive selection during a genome-wide scan of human and primate DNA for signs of adaptive evolution. The current study examined the hypothesis that a single-nucleotide polymorphism (SNP) C14625T (rs7671281) in the *ENAM* gene identified in the genome-wide scan is associated with a change in enamel phenotype. African Americans were selected as the target population, as they have been reported to have a target SNP frequency of approximately 50%, whereas non-Africans are predicted to have a 96% SNP frequency. Digital radiographs and DNA samples from 244 teeth in 133 subjects were analysed, and enamel thickness was assessed in relation to SNP status, controlling for age, sex, tooth number and crown length. Crown length was found to increase with molar number, and females were found to have thicker enamel. Teeth with larger crowns also had thicker enamel, and older subjects had thinner enamel. Linear regression and generalized estimating equations were used to investigate the relationship between enamel thickness of the mandibular molars and *ENAM* SNP status; enamel in subjects with the derived allele was significantly thinner (*P*=0.040) when the results were controlled for sex, age, tooth number and crown length. The derived allele demonstrated a recessive effect on the phenotype. The data indicate that thinner dental enamel is associated with the derived *ENAM* genotype. This is the first direct evidence of a dental gene implicated in human adaptive evolution as having a phenotypic effect on an oral structure.

## Introduction

Teeth are crucial for animal survival, and the preservation of their function is important in natural selection.^1^ Molar enamel thickness has been utilized to study relationships among primates and hominoids and has been considered an important factor in the adaptation of the dentition to specific diets.^2^ Measurements of enamel thickness in primates and hominoids have shown that thinner enamel is associated with a diet that is primarily foliage or fruits and thicker enamel is associated with a diet of harder objects, such as seeds and grains, that require crushing and grinding.^3, 4^ Among related forms of primates, those consuming softer foods have thinner enamel than those that eat harder foods.^5^ Evidence of positive selection has been found in *ENAM* along the lineage leading to chimpanzees,^6^ and thicker enamel is the ancestral condition of the great apes.^4^

Data on human teeth from as far back as the genus Homo for the genus *Homo* in Africa, ~2.5 million years ago, reveal changes in enamel thickness, possibly due to dietary change.^4, 7^ Cultural changes might have led to a more recent, rapid adaptation in the human genome as diet changed from hard objects, such as seeds and nuts, to include domesticated animals and plants; in addition, the development of cooking enabled increased intake of high-calorie, nutrient-dense foods.^8^ As the diet of early humans changed from harder objects to meat and cooked foods, thinner enamel might have been advantageous in maintaining sharp enamel crests, which are better for shearing, versus thicker enamel, which promotes resistance to grinding,^9^ or perhaps there was less selective pressure to maintain thicker enamel.

The enamelin (*ENAM*) gene encodes an enamel matrix protein that is essential to the formation of tooth enamel. It is located on chromosome 4:71859495-71877517, reference sequence NM_031889 (UCSC Genome Browser March 2006 Assembly).^10^ Enamelin is the largest enamel protein, a 1142 amino acid secretory protein with a 39 amino acid signal peptide.^11^ During enamel formation, the protein is found among the developing crystallites in the rod and interrod enamel. The physical properties of enamel depend on the expression of *ENAM* along with the amelogenin and ameloblastin genes. Although the exact function of enamelin is not well understood, it is thought to contribute to the elongation of crystals in the developing enamel.^12^ Enamel crystals form perpendicularly to the outer surface of the tooth, consistent with a contribution of the ENAM protein to thicker enamel.

*ENAM* in humans was found to be subject to possible (*P*=0.053) positive selection using an outlier approach in a genome-wide scan of European Americans.^13^ Further exploration of the target led to the identification of a non-synonymous derived allele (SNP C14625T, rs7671281) that might be correlated with adaptive evolution in humans. This allele occurs at a frequency of 26.9% in a sub-Saharan African population and is nearly fixed in non-African populations at a frequency of 96.5%.^10^ The SNP changes the amino acid 648 from threonine to isoleucine, changing a polar side-chain to a non-polar side-chain. It is unclear how this change affects the three-dimensional structure of the protein, but it is thought to potentially affect protein function. American blacks have been shown to have larger-diameter permanent teeth than American whites.^14^ In addition, enamel thickness varies between American blacks and American whites in the primary dentition, even when controlled for tooth size.^14, 15^ We hypothesized that the non-synonymous SNP accounts for these differences.

Despite findings from the genome-wide scan demonstrating a change in the genotype of *ENAM*, to date no data exist that show the phenotypic effects of the polymorphism. The current study investigated the phenotypic effect of SNP C14625T (rs7671281) on the thickness of human dental enamel, exploring the effects of positive selection on a dental gene.

## Materials and methods

### Samples

Approval for this study was obtained from the Human Subjects Division at the University of Washington, and all subjects provided written informed consent. African-American subjects aged 18–75 who had a least one permanent mandibular molar without visible caries or restorations were enrolled. No more than one subject was eligible per extended family. A buccal swab DNA sample and four bitewing radiographs were taken for each subject. African Americans were selected as the study population to allow a greater chance of relatively equal distribution of the ancestral allele and derived allele among the subjects. The determination of African-American status was based on the subjects' self-identification. Age and sex were recorded and included for statistical analysis.

### Radiographs

An intraoral x-ray imaging system (Heliodent DS, Sirona, Benshein, Germany) and phosphor imaging plates (OpTime, Soredex, Milwaukee, WI, USA) were used to capture digital dental radiographs of the subjects, with settings of 60 kVp, 7 mA, and 0.32 s. Two similar radiographs were taken of both the right and left side to ensure an image without overlap of the inter-proximal contacts but with maximal overlap of the buccal and lingual cusps, to ensure parallelism and avoid distortion of the image. The radiographs were processed in an intraoral digital imaging system (OpTime, Soredex, Milwaukee, WI, USA). All radiographs were taken by experienced clinicians. Bitewing radiographic images were evaluated by a team of examiners to ensure that they met the required selection criteria.

### Enamel Thickness Measurement

Because the first, second, and third mandibular molars vary in size,^16, 17^ we initially selected bilateral mandibular first molars for measurement. If the first molar was not available, the second or third molar was selected and measured, based on availability. Tooth number was therefore included as a “within subject variable” of the generalized estimating equation (GEE). Molar enamel thickness depends on the size of the teeth^15^ and on sex.^9, 16^ Therefore, the subjects' sex and crown length were included as covariates.

**Figure 1:**
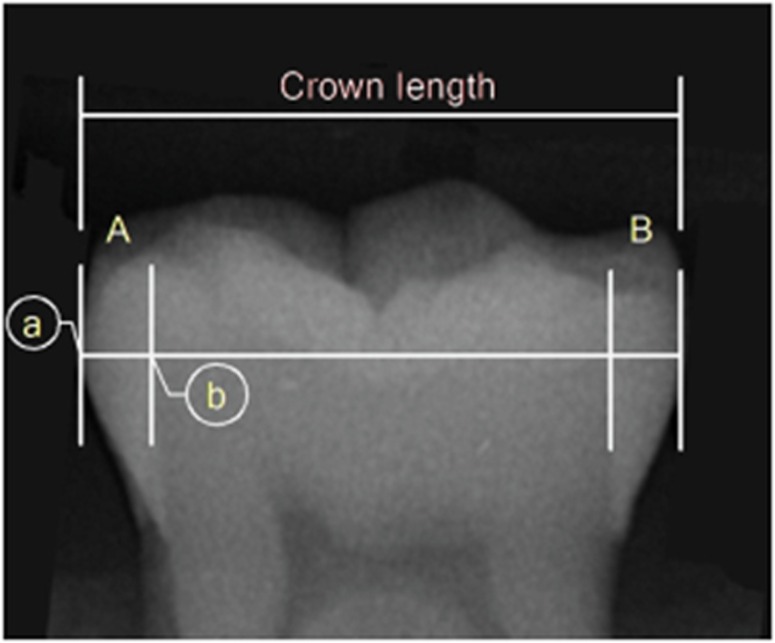
**Radiographic tooth measurements**. Crown length is the distance from the mesial height of contour to the distal height of contour. A and B are the mesial and distal enamel thickness measured from a to b; a=height of contour, b=the point where the crown length crosses the dentino-enamel junction.

In each digital radiograph (Figure 1), vertical lines were drawn at the mesial and distal heights of contour. A line was drawn connecting the mesial and distal heights of contour, and this was used as the crown length. The thickness of the enamel on the mesial (A) and distal (B) surfaces was measured from the height of contour to the point where the line connecting the mesial and distal heights of contour crossed the dentino–enamel junction. The measurement was made in millimetres using two-dimensional design measurement software (Autodesk Design Review 2013; Autodesk, San Rafael, CA, USA).

The scale of the radiographs was determined by measuring the height and width of the digital x-ray imaging plate with an electronic dental calliper. The plate was then used to determine the scale of the radiograph once images were uploaded into the design review software. One individual (Y.G.U.) performed all of the measurements to minimize error and maintain consistency. To determine the reliability of tooth measurements, we took a random sample of 20 teeth and the same investigator (Y.G.U.) measured them three times on different dates in random order, for a result of 97%±2% reliability.

### DNA Collection and Analysis

Genomic DNA was isolated from the study population by extracting oral cytobrush samples using a DNA extraction kit (Epicentre Technologies, Madison, WI, USA) according to the manufacturer's instructions. The samples were collected and frozen prior to processing. Polymerase chain reaction (PCR) gene amplification was performed according to a method described by Kelley and Swanson.^10^ The primers and conditions for PCR and sequencing are available upon request. All reactions were carried out in microtiter plates using multichannel pipettes, which made it feasible to scale to multiple individuals and decreased the likelihood of error due to sample processing. The PCR products were cycle sequenced directly using BigDye v. 3.1 (Applied Biosystems, Foster City, CA, USA), ethanol precipitated, and analysed on an automated sequencer (ABI 3100; Applied Biosystems, Foster City, CA, USA). Base calling and contig assembly were done using Phred/Phrap^18, 19^ and visually inspected with Consed.^20^ Polymorphic sites were automatically identified by PolyPhred^21^ and verified manually.

### Data Analysis

A linear regression was used with a recessive model to investigate the influence of SNP status in on enamel thickness, controlling for sex, age, and tooth number and site, with an alpha of 0.05. To account for clustering of teeth, we performed modelling with a GEE, employing a Wald *χ*^2^-test for testing model effects using subject number as the subject identifier and tooth number and tooth surface as the within-subject variables. The relationship of sex to tooth size and enamel thickness was determined using a *t*-test. The statistics were calculated on a personal computer utilizing statistical software (IBM SPSS Statistics 19; IBM, Armonk, NY, USA).

## Results

### Study Population

Enrolment and study procedures were completed for 195 subjects. Individuals missing mandibular molars or having caries or restorations were excluded from the study, as were those for whom overlapping images did not allow accurate measurement of the mandibular molars. Ultimately, radiographs for 148 subjects were selected by a team that was blinded to SNP status. There was a 10% DNA extraction failure rate; DNA extractions were successful for 133 of 148 subjects. The majority of the subjects with successful DNA extractions had two measurable mandibular molars. Of the 133 subjects with both measurable radiographs and successful DNA extraction, 22 subjects (11 male and 11 female) were included in the analysis with only one measurable molar. The data analysis was based on a total of 244 total measurable molars. The SNP status of our sample was very close to the expected frequency for an African-American population, with resulting genotype frequencies of 25% C/C, 51.5% C/T and 23.5% T/T, where C is the ancestral allele; the genotypes were found to be in Hardy–Weinberg equilibrium.

### Enamel Thickness Data by Covariates

**Figure 2:**
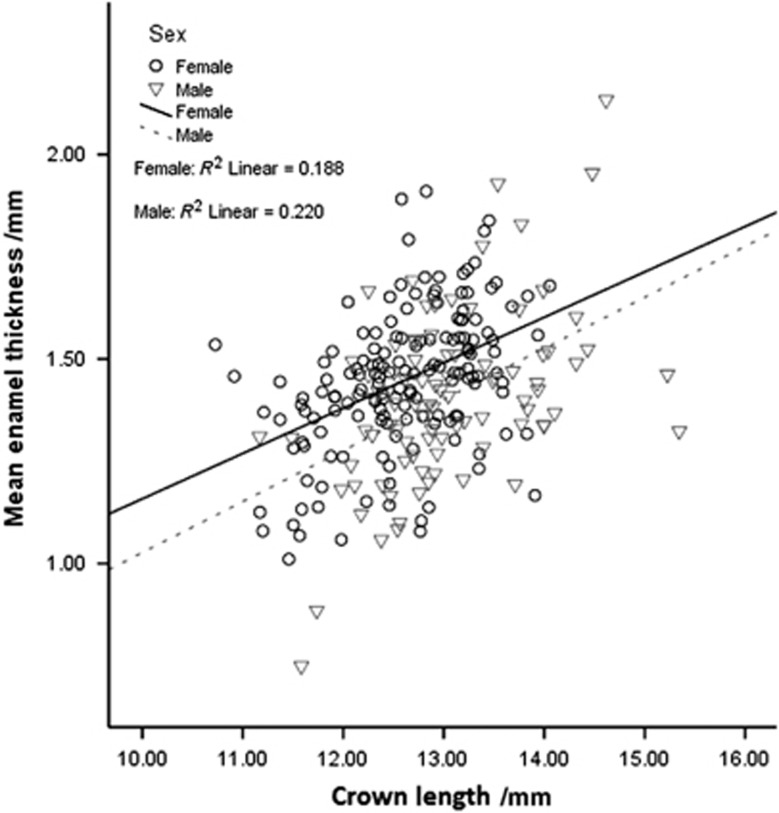
**Correlations in enamel thickness to crown length and sex**. When controlled for sex, average enamel thickness increases with crown length (*P*<0.001).

Descriptive statistics for the molars that were measured are provided in Table 1. Because enamel thickness can vary depending on age, sex, and tooth size, the effect of each variable was first examined independent of SNP status. Sex differences were analysed first. Without normalizing for crown length, females had significantly (*P*=0.031) thicker enamel than males, and males had significantly longer crowns (*P*<0.001). To determine whether larger teeth had thicker enamel in males and females, we investigated crown length in relation to average enamel thickness. Larger teeth were found to correlate with thicker enamel (Figure 2). When controlling for the smaller teeth in females compared with males, females still had significantly (*P*<0.001) thicker enamel.

**Figure 3:**
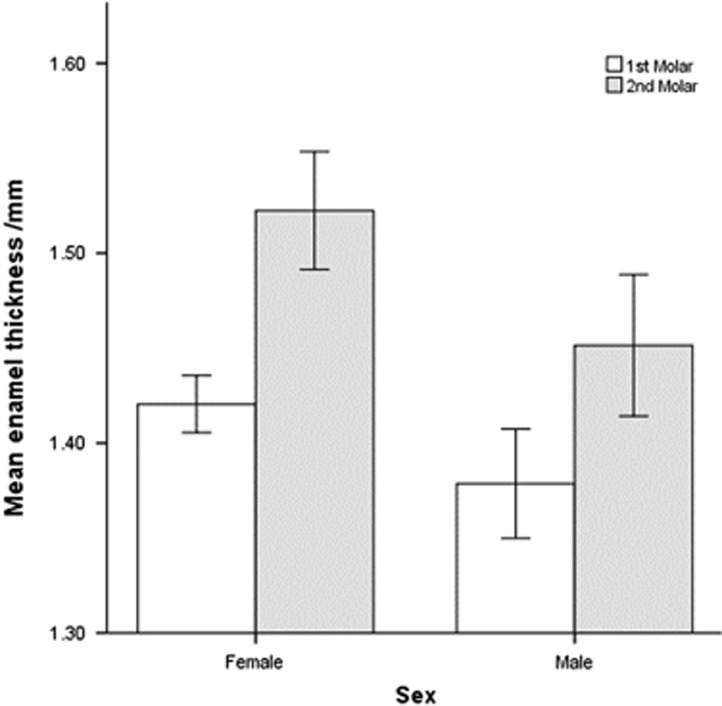
**Enamel thickness by sex and molar number.** Enamel thickness increased from the 1st to the 2nd molar (*P*<0.001). Error bars±1 standard error.

Mesial enamel thickness tended to increase slightly from the first molar to the second molar when controlled for sex. It also increased from the second to third molar in males. The female sample for third molars included only one tooth, which was not included in the analysis. Distal enamel thickness significantly (*P*<0.01) increased from the first molar to the second molar when controlled for sex. It also increased from the second to third molars in males. In addition, enamel thickness increased from the first to second molar (*P*<0.001; Figure 3).

### SNP Status and Enamel Thickness

**Figure 4:**
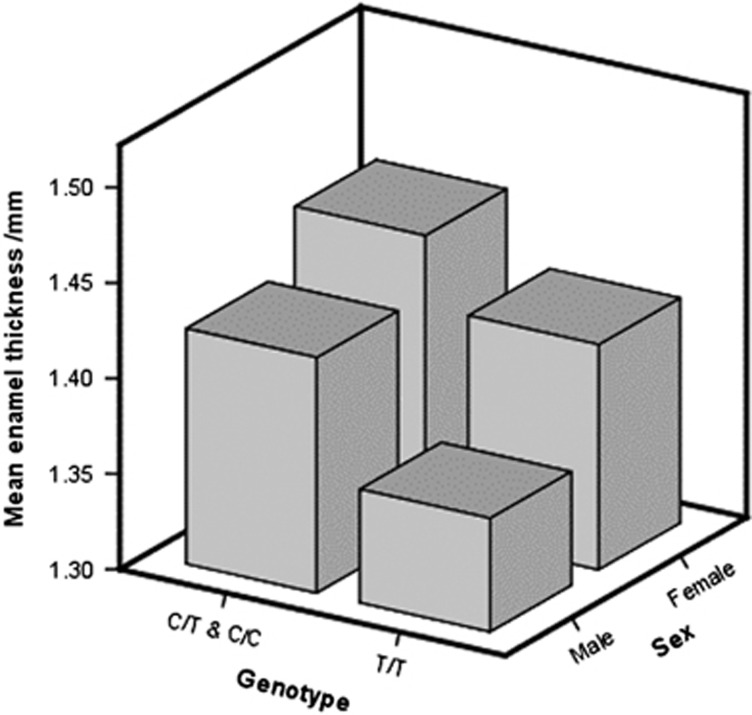
**Distribution of enamel thickness by genotype and sex.** The enamel is thinner in subjects with two copies of the derived allele (C/C) (*P*=0.04).

An analysis of enamel thickness by both genotype and sex revealed that, for both males and females, any copy of the ancestral allele was associated with thicker enamel, and females had thicker enamel than males. This was found for average enamel thickness, even in the absence of normalizing for differences in crown length. When crown length was taken into account using linear regression with a GEE model, the enamel was thinner for both males and females when two copies of the derived allele were present (*P*=0.04; Figure 4).

### SNP Recessive Model

**Figure 5:**
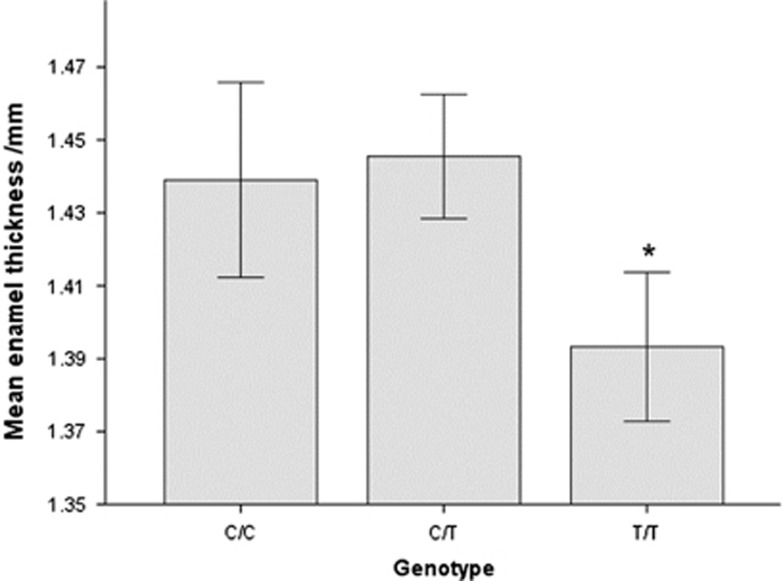
**Analysis of enamel thickness by genotype.** Subjects with two copies of the derived allele (T/T) have significantly (*P*=0.04) thinner enamel. Error bars±1 standard error.

Subjects with two copies of the derived allele (SNP C14625T) had significantly (*P*=0.04) thinner enamel when the data were controlled for molar number, tooth size, age, and sex and accounting for clustering of teeth and tooth surfaces using the GEE. Figure 5 summarizes the findings, which support a recessive model for the mutation C14625T. This analysis also found that wider teeth and teeth from females had significantly thicker enamel (*P*<0.001 and *P*<0.01, respectively). Although older subjects tended to have thinner enamel, this difference was not significant (*P*=0.07). With respect to molar number (first, second, or third), of the 171 first molars, 46 (27%) were from T/T subjects; of the 65 s molars, 9 (14%) were T/T; of the 7 third molars, 2 (28%) were T/T. If only first molars were considered, the molars from subjects with the homozygous-derived allele T/T tended to have thinner enamel, but the difference was not significant (data not shown).

## Discussion

Our data confirm previous findings that females have thicker enamel, enamel thickness is greater on larger teeth, molars' enamel thickness increases with molar number and enamel thickness increases when going from first to second to third molars. The confirmation of previous findings also demonstrates the reliability of the method for measuring enamel thickness. Our analysis was based on an *a priori* decision to control for sex, age, molar number, and crown length due to these previous studies. The data in Figure 4 show, however, that even without controlling for these covariates, subjects with both copies of the derived allele had thinner enamel.

Consistent with the hypothesis that changes in *ENAM* can lead to thinner enamel, mutations in *ENAM* have previously been shown to cause several types of amelogenesis imperfecta, disorders that affect the formation of dental enamel and cause hypoplastic enamel that is thinner and softer than that of normal teeth.^22, 23^ Mutations have been found to have both autosomal dominant and autosomal recessive patterns of inheritance, with the phenotype of the *ENAM* mutations possibly being dosage-dependent and ranging from general hypoplasia to localized enamel pitting.^24^ Typically, the enamel is thinner than normal.^11, 22^

The ancestral (C) allele of the SNP C14625T is present at a much higher frequency in African populations than in non-African populations. We did not ascertain the exact origin of our subjects using ancestry-informative markers,^25^ which is a limitation of the study and could make it vulnerable to false positives due to population stratification/admixture; however, the recruited African-American subjects were predicted to have a 50% target SNP frequency and were found to meet this target, which enabled us to examine the effects of the SNP in a single population.

Gene–culture interactions have been documented to be responsible for faster and stronger evolutionary dynamics and might be the dominant form of human evolution.^26^ Dietary changes and thinning enamel are an example of a gene–culture interaction. This study expands on the findings of adaptive evolution demonstrated by the non-synonymous-derived allele (rs7671281) and provides direct evidence relating it to thinner enamel. Although it is believed that thinner enamel is advantageous for shearing, thinner enamel might not be an advantage with a modern diet that is much higher in refined carbohydrates. Dental caries was not a common finding in early humans^27^ because of their diet low in refined carbohydrates; therefore, evolutionary pressure for thinner enamel would not have been an adverse consequence. It has been suggested that the influence of genetic variation of enamel formation genes may influence susceptibility to caries.^28^ A recent study looked at *ENAM* variants and caries susceptibility and found that none of the evaluated SNPs, which were studied separately, were associated with caries when classic caries risk factors were included in the model; however, when rs7671281 was analysed in the presence of another *ENAM* SNP (rs3796704), caries susceptibility increased 2.66-fold, independent of other risk factors.^29^ This increased caries risk might be due to the thinner enamel of the derived allele. Dental caries is a complex disease with many associated risk factors but may be, at least in part, a modern consequence of the SNP C14625T and thinner enamel. This is the first direct evidence of a role of a dental gene implicated in the adaptive evolution of the human species.

## Figures and Tables

**Table 1:** Tooth properties by sex

Sex	Mesial enamel thickness /mm	Distal enamel thickness /mm	Average enamel thickness /mm	Crown length M–D /mm	Age
Male					
Mean	1.379	1.434	1.406	13.041	32
s.e.	0.023	0.026	0.022	0.082	1.2
Female					
Mean	1.406	1.487*	1.447*	12.599**	30
s.e.	0.016	0.017	0.014	0.055	0.9

s.e., standard error.

Significantly different from male *P<0.05, **P<0.001 (*t*-test).

Men have significantly longer crowns; women have significantly thicker mean enamel.
